# Prevalence of fosfomycin resistance and gene mutations in clinical isolates of methicillin-resistant *Staphylococcus aureus*

**DOI:** 10.1186/s13756-020-00790-x

**Published:** 2020-08-17

**Authors:** Yi-Chien Lee, Pao-Yu Chen, Jann-Tay Wang, Shan-Chwen Chang

**Affiliations:** 1 Department of Internal Medicine, Fu Jen Catholic University Hospital, Fu Jen Catholic University, New Taipei City, Taiwan; 2 School of Medicine, College of Medicine, Fu Jen Catholic University, New Taipei City, Taiwan; 3 Department of Internal Medicine, National Taiwan University Hospital, 7 Chung-Shan South Road, 100 Taipei, Taiwan; 4 Institute of Infectious Diseases and Vaccinology, National Health Research Institutes, Tsu-Nan County, Taiwan

**Keywords:** Fosfomycin, Resistance, Gene mutations, Methicillin-resistant *Staphylococcus aureus*

## Abstract

**Background:**

Fosfomycin exhibits excellent in vitro activity against multidrug-resistant pathogens, including methicillin-resistant *Staphylococcus aureus* (MRSA). Increasing fosfomycin resistance among clinical MRSA isolates was reported previously, but little is known about the relative abundance of Fosfomycin resistance genes in MRSA isolates circulating in Taiwan.

**Methods:**

All MRSA isolates, collected in 2002 and 2012 by the Taiwan Surveillance of Antimicrobial Resistance (TSAR) program, were used in this study. Susceptibility to various antimicrobial agents, including fosfomycin, was determined by broth microdilution. Genetic determinants of fosfomycin resistance, including *fosB* carriage and *murA*, *glpT* and *uhpT* mutations, were investigated using PCR and sequencing of amplicons. Staphylococcal protein A (*spa*) typing was also performed to determine the genetic relatedness of MRSA isolates.

**Results:**

A total of 969 MRSA strains, 495 in the year 2002 and 474 in the year 2012, were analyzed. The overall in vitro susceptibility was 8.2% to erythromycin, 18.0% to clindamycin, 29.0% to tetracycline, 44.6% to ciprofloxacin, 57.5% to trimethoprim/sulfamethoxazole, 86.9% to rifampicin, 92.9% to fosfomycin and 100% to linezolid and vancomycin. A significant increase in the fosfomycin resistance rate was observed from 3.4% in 2002 to 11.0% in 2012. Of 68 fosfomycin-resistant MRSA isolates, several genetic backgrounds probably contributing to fosfomycin resistance were identified. Twelve isolates harbored the *fosB* gene, and various mutations in *murA*, *uhpT*, and *glpT* genes were noted in 11, 59, and 66 isolates, respectively. The most prevalent gene mutations were found in the combination of uhpT and glpT genes (58 isolates). The vast majority of the fosfomycin-resistant MRSA isolates belonged to *spa* type t002.

**Conclusions:**

An increased fosfomycin resistance rate of MRSA isolates was observed in our present study, mostly due to mutations in the *glpT* and *uhpT* genes. Clonal spread probably contributed to the increased fosfomycin resistance.

## Background

Fosfomycin, a phosphonic acid derivative produced by *Streptomyces* spp. and discovered in 1969 [1], displays broad-spectrum activity against both Gram-negative and Gram-positive pathogens. It is a bactericidal antimicrobial agent that interferes with the enzyme-catalyzed bacterial cell wall synthesis [2]. Numerous studies have demonstrated the excellent in vitro susceptibility of multidrug-resistant and extensively drug-resistant organisms (MDRO and XDROs) to fosfomycin, including vancomycin-resistant enterococci (VRE) (96%) [3], ESBL-producing *Enterobacteriaceae* (87.7%) [4], carbapenem-resistant Gram-negative bacteria (99%) [5], and methicillin-resistant *Staphylococcus aureus* (MRSA) (99.6%) [6]. Additionally, the synergistic effect of fosfomycin in combination with other relevant antibiotics against the above-mentioned MDR microorganisms, evaluated by time-kill experiments, checkerboard analysis and E-test methods [7–9], was promising. These studies indicated that fosfomycin could be a potential treatment option for the difficult-to-treat infections caused by drug-resistant organisms.

Among MDROs, MRSA is a major human pathogen which causes various dangerous infections, such as bacteremia, endocarditis, and abscess, in both community and hospital settings [10]. Fosfomycin alone or combined with other antimicrobial agents exhibited favorable in vitro activity against MRSA [6, 8, 11, 12], and more than 70% clinical cure was observed with fosfomycin administration for the treatment of MRSA infection [13, 14]. However, *S. aureus* with fosfomycin resistance developed and rose rapidly by 30–70% in China [15]. The mechanism of bacterial resistance to fosfomycin could involve either a chromosome-associated defective transport system or plasmid-mediated fosfomycin-inactivating enzymes. First, two key transporter systems, GlpT and UhpT, mediated the entry of fosfomycin into bacterial cells [16]. Once mutations in the chromosomal *glpT* and *uhpT* genes occurred, reduction in permeability with subsequent conferred MRSA resistance to fosfomycin was observed [16–18]. Second, the *murA* gene mutants exhibited lower affinity for fosfomycin [19], conferring various degrees of drug resistance. Moreover, a few fosfomycin-modifying enzymes, including FosA, FosB, FosC, and FosX, catalyzed the inactivation of fosfomycin [20–22], and only FosB is produced by Gram-positive bacteria [23]. Most of the previous studies investigated the mechanism of fosfomycin resistance among Gram-negative bacteria, and only limited information about the resistance mechanism of Gram-positive pathogens, particularly MRSA, is available. Hence, in the present study, we aimed to survey the prevalence of fosfomycin resistance and the associated *uhpT, glpT, murA*, and *fosB* genetics in clinical isolates of MRSA in Taiwan.

## Methods

### Bacterial isolates

All MRSA strains, collected in 2002 and 2012 through the TSAR program from different hospitals in Taiwan, were used in this study. The principles of isolate collection by the TSAR program had been described clearly in a previous study [8]. Duplicate isolates were excluded, and a total of 969 MRSA isolates, 495 collected in 2002 and 474 in 2012, were analyzed. All these strains were identified as *S. aureus* by performing Gram staining, a catalase-activity test, and a coagulase latex agglutination test (automated VITEK-2 system, Biomerieus, France). Methicillin resistance was ascertained using agar disk diffusion (Kirby-Bauer), according to the guidelines established by the Clinical and Laboratory Standards Institute (CLSI) [24]. The study was approved by the Ethical Committee of the National Taiwan University Hospital (NTUH-IRB No. 201504056RINB).

### Antimicrobial susceptibility

The antimicrobial susceptibility to clindamycin, ciprofloxacin, erythromycin, linezolid, rifampicin, trimethoprim/sulfamethoxazole, tetracycline, fosfomycin, and vancomycin was determined using a broth microdilution method according to the CLSI recommendations [24], and the results were interpreted using the criteria for *S. aureus* provided by the CLSI [24]. *Staphylococcus aureus* ATCC 29213 was used as the internal control for each run of the susceptibility test.

### Genetic analysis

DNA of 68 fosfomycin-resistant MRSA isolates was harvested using a DNA Extraction System kit (Viogene, New Taipei City, Taiwan) according to the manufacturer’s instructions. The presence of *fosB* was detected by PCR using the previously described primers [25], and the full nucleotide sequence of three genes (*murA, uhpT,* and *glpT*) was determined by combing direct sequencing and primer walking with the individual PCR products. Primers, used in the present study, are shown in Table 1. The PCR and sequencing procedures were described in prior studies [15, 25].

**Table 1:** PCR primers of *fosA, fosB, fosC, murA, glpT*, and *uhpT* genes

Primers	Genes	Primer sequences (5 > 3)	Product size	References
fosB-F	fosB	CAGAGATATTTTAGGGGCTGACA	312 bp	[25]
fosB-R		CTCAATCTATCTTCTAAACTTCCTG		
murA-F	murA	GCCCTTGAAAGAATGGTTCGT	1600 bp^a^	NC_002745.2^b^
murA-R		GTTACAATACTCGACGCAGGT		
glpT-F	glpT	TGAATAAAACAGCAGGGCAA	1699 bp^a^	NC_002745.2^b^
glpT-R		CACAGCTAGTATGTATAACGAC		
uhpT-F	uhpT	TGTGTTTATGTTCAGTATTTTGGA	1571 bp^a^	NC_002745.2^b^
uhpT-R		TCTTTCATCTCTTCACGCAC		

^a^ PCR product including surrounding sequences adjacent to target gene

^b^ GenBank-EMBL-DDBL accession number

### Molecular typing

Staphylococcal protein A (*spa* gene) typing was performed for 68 fosfomycin-resistant MRSA isolates. The highly variable X region in *spa* was amplified by PCR using the previously described primers [26]. The purified PCR products were sequenced, and the *spa* types were confirmed by analyzing the nucleotide sequence utilizing BioNumerics Version 6.5 (Applied Maths NV, Sint-Martens-Latem, Belgium) [27].

### Statistical analysis

Categorical variables were expressed as percentages, and Fisher’s exact test with two-sided comparison was utilized for the assessment of statistical significance.

## Results

### Susceptibilities to tested antimicrobial agents

The overall susceptibilities to various antibiotics was demonstrated in Table 2. Susceptibility rates to clindamycin, erythromycin, tetracycline, and trimethoprim/sulfamethoxazole increased from 2002 to 2012; however, the susceptibility rates to rifampicin and fosfomycin between 2002 and 2012 decreased statistically significantly, shown in Table 2. All tested MRSA isolates remained 100% susceptible to linezolid and vancomycin. Among the fosfomycin-resistant MRSA isolates, lower susceptibility rates to clindamycin, ciprofloxacin, erythromycin, and rifampicin were noted compared with those of the fosfomycin-susceptible group. In contrast, the susceptibility rates to trimethoprim/sulfamethoxazole and tetracycline within the fosfomycin-susceptible group was lower (Table 3).

**Table 2:** Antibiotics susceptibilities grouped by study year

Antibiotics^a^	Overall susceptibilities (%) (*n* = 968)	Susceptibilities by years (%)		
		2002 (*n* = 495)	2012 (*n* = 473)	*P*
Clindamycin	174 (18.0)	49 (9.9)	125 (26.4)	< 0.001
Ciprofloxacin	432 (44.6)	224 (45.3)	208 (44.0)	0.699
Erythromycin	79 (8.2)	17 (3.4)	62 (13.1)	< 0.001
Linezolid	968 (100)	495 (100)	473 (100)	–
Rifampicin	841 (86.9)	456 (92.1)	385 (81.4)	< 0.001
SXT	557 (57.5)	236 (47.7)	321 (67.9)	< 0.001
Tetracycline	281 (29.0)	70 (14.1)	211 (44.6)	< 0.001
Fosfomycin	900 (93.0)	478 (96.6)	422 (89.2)	< 0.001
Vancomycin	968 (100)	495 (100)	473 (100)	–

^a^ Antibiotic abbreviation: SXT, trimethoprim/sulfamethoxazole

**Table 3:** Susceptibilities of different antibiotics in methicillin-resistant *Staphylococcus aureus* isolates divided by sensitive to fosfomycin and resistance to fosfomycin

Antibiotics^a^	Susceptibilities by (%)		Statistics
	Fosfomycin-Susceptible (*n* = 900)	Fosfomycin-Resistant (*n* = 68)	P
Clindamycin	173 (19.2)	1 (1.5)	< 0.001
Ciprofloxacin	431 (47.9)	1 (1.5)	< 0.001
Erythromycin	79 (8.8)	0 (0)	0.005
Linezolid	900 (100)	68 (100)	–
Rifampicin	833 (92.6)	9 (13.2)	< 0.001
SXT	495 (55.0)	62 (91.2)	< 0.001
Tetracycline	221 (24.6)	60 (88.2)	< 0.001
Vancomycin	900 (100)	68 (100)	–

^a^ Antibiotic abbreviation: SXT, trimethoprim/sulfamethoxazole

### Prevalence of fosfomycin resistance genes

Patients with fosfomycin-resistant MRSA colonization/infection have a median age of 79 years (interquartile range: 69–85), which is significantly elder than that of patients with fosfomycin-susceptible MRSA (median, 59; interquartile range: 33–76). Most of those fosfomycin-resistant MRSA isolates were collected from the hospitals located in central (37 isolates) or southern (20 isolates) Taiwan, but the majorities of fosfomycin-susceptible MRSA strains were isolated from central (34.9%) and northern Taiwan (28%). Of 68 fosfomycin-resistant MRSA isolates, 12 strains harbored the *fosB* gene with fosfomycin MICs ranging from 128 to > 2048 mg/L (Table 4). Classification of different mutations in the murA, uhpT, and glpT genes, including various nucleic acid deletions and amino acid substitutions, was defined as following: type ImurA, G257D; type IImurA, D278E; type IIImurA, deletion at position 717; type IVmurA, G322S; type VmurA, L162I; type VImurA, E271Q; type VIImurA, G240R; type IuhpT, A279V; type IIuhpT, A297V/E225D; type IIIuhpT, F267L/L281X; type IVuhpT, G161R; type IglpT, A434V; type IIglpT, W147X; type IIIglpT, F197I; type IVglpT, A434V/G399S; type VglpT, C57X; type VIglpT, T313K. Nevertheless, a total of 11 isolates expressed a *murA* mutation, and seven different subtypes, including type I_*murA*_ to type VII_*murA*_, were identified in these mutant genes. Only type III_*murA*_ led to a deletion of one nucleic acid at position 717, and the other six mutations (type I-II_*murA*_ and type IV-VII_*murA*_) resulted in distinct amino acid substitutions within the MurA protein. The most commonly encountered fosfomycin-resistant *murA* mutant was type I (4 isolates).

**Table 4:** Distributions of fosfomycin-resistant related genes corresponding MIC values among MRSA isolates

*uhpT* mutation^a^	*glpT* mutation^b^	*murA* mutation^c^	*fosB* gene	t002		t037		Other *spa* types		Total	
				n	FOS range	n	FOS range	n	FOS range	n	FOS range
Wild type	Wild type	Wild type	Positive	0	–	0	–	1	128	1	128
Wild type	Type I	Wild type	Positive	1	> 2048	0	–	0	–	1	> 2048
Wild type	Type I	Wild type	Negative	3	> 2048	0	–	1	> 2048	4	> 2048
Wild type	Type II	Type I	Negative	0	–	2	> 2048	0	–	2	> 2048
Wild type	Type VI	Wild type	Negative	0	–	1	64	0	–	1	64
Type I	Wild type	Type I	Negative	0	–	1	128	0	–	1	128
Type I	Type I	Wild type	Positive	9	> 2048	0	–	0	–	9	> 2048
Type I	Type I	Wild type	Negative	31	1024 - > 2048	0	–	7	> 2048	38	1024 - > 2048
Type I	Type I	Type III	Positive	1	> 2048	0	–	0	–	1	> 2048
Type I	Type I	Type IV	Negative	2	> 2048	0	–	0	–	2	> 2048
Type I	Type I	Type V	Negative	1	> 2048	0	–	0	–	1	> 2048
Type I	Type I	Type VII	Negative	1	> 2048	0	–	0	–	1	> 2048
Type I	Type III	Wild type	Negative	1	128	0	–	0	–	1	128
Type I	Type IV	Wild type	Negative	1	> 2048	0	–	0	–	1	> 2048
Type II	Type I	Wild type	Negative	1	> 2048	0	–	0	–	1	> 2048
Type II	Type I	Type VI	Negative	0	–	0	–	1	> 2048	1	> 2048
Type III	Type I	Type II	Negative	0	–	0	–	1	256	1	256
Type IV	Type V	Type I	Negative	0	–	1	2048	0	–	1	2048

^a^ Type I: A279V; Type II: A297V/E225D; Type III: F267L/L281X; Type IV: G161R; Wild type: no mutations detected

^b^ Type I: A434V; Type II: W147X; Type III: F197I; Type IV: A434V/G399S; Type V: C57X; Type VI: T313K; Wild type: no mutations detected

^c^ Type I: G257D; Type II: D278E; Type III: deletion at position 717; Type IV: G322S; Type V: L162I; Type VI: E271Q; Type VII: G240R; Wild type: no mutations detected

Sixty-six of 68 fosfomycin-resistant MRSA strains contained one of the six different mutations (type I-VI_*glpT*_) found in the *glpT* gene with the majority containing a type I_*glpT*_ mutation (60 isolates). Each of these mutations caused amino acid substitutions within the GlpT protein. Furthermore, four different mutations (type I-IV_*uhpT*_) were recognized in the *uhpT* gene of the 59 fosfomycin-resistant MRSA isolates with type I_*uhpT*_ as the most prevalent (55 isolates). Similarly, the four mutations resulted in amino acid substitutions within the UhpT protein. Likewise, 58 fosfomycin-resistant MRSA isolates displayed dual resistance mechanisms. The details of those fosfomycin-resistant MRSA isolates harboring different types of mutation genes were described in Table 4.

### Molecular typing

The 68 fosfomycin-resistant MRSA strains were classified into several *spa* types, including t002 (52 isolates), t037 (5 isolates), and other *spa* types. The 52 *spa* t002 fosfomycin-resistant isolates had a greater proportion (51/52) of high fosfomycin MICs (1024 ~ > 2048) than in the other 16 (12/16) fosfomycin-resistant isolates (*p* = 0.019). Among them, 11 isolates harbored the *fosB* gene; 5 strains, the *murA* gene; 52 and 48 mutants, the *glpT* and *uhpT* genes, respectively.

## Discussion

A unique mechanism of action of fosfomycin made cross-resistance to other antibiotic classes less common, which motivated physicians to reevaluate its ability to destroy drug-resistant pathogens, including MRSA [16]. In our study, elderly patients seemingly had the tendency of acquisition of fofsomycin-resistant MRSA infections, and the vast majority of those resistant strains were isolated from those hospitals located in central or southern Taiwan. The background mechanisms of this phenomenon need further investigation. Although the MRSA isolates exhibited high in vitro susceptibility to fosfomycin higher than 90%, a significant increase in fosfomycin resistance rate during past decades (from 3.4% in 2002 to 11.0% in 2012) was observed in Taiwan. Among the fosfomycin-resistant isolates, a higher resistance rate to clindamycin, ciprofloxacin, erythromycin, and rifampicin was noted; however, trimethoprim/sulfamethoxazole and tetracycline displayed more favorable susceptibility. Type I_*uhpT*_ and type I_*glpT*_ mutations predominantly caused fosfomycin resistance in our MRSA isolates, and the vast majority of isolates belonged to *spa* type t002.

Little was known about the MRSA fosfomycin resistance mechanism in epidemiological research, and very few literature reports were previously published [15, 28, 29]. Of those studies, a large-scale surveillance conducted by Fu et al. [15] in China demonstrated a 13.4% (9/67) *fosB*-positive rate with two-thirds (6/9) belonging to ST5. A research study by Etienne et al. [28] revealed that 18 of 39 (46.2%) *S. aureus* isolates, containing the *fosB* gene, were highly resistant to fosfomycin, but only one MRSA isolate had the *fosB* gene (Zhejiang, China) [29]. In our present study, approximately one-fifth of the MRSA isolates with fosfomycin resistance carried the *fosB* gene with the dominant t002 *spa* typing (11/12), which belonged to ST5. Our finding was similar to that described in Fu’s report; it implicated the presence of clonal spread among the *fosB*-positive MRSA isolates, despite the previously reported triviality of *fosB* [30].

MurA, a target enzyme involved in the biosynthesis of bacterial peptidoglycan, could be inactivated by fosfomycin via its binding to the active site of the enzyme [16]. However, mutations of the *murA* gene resulted in amino acid substitutions, rendering susceptible clinical isolates resistant to fosfomycin [16]. Fu et al. [15] illustrated that a *murA* mutation played an unclear role in the fosfomycin resistance in their study, and a type II_*murA*_ mutant was the most common among all *murA* mutations. Our results were different in this regard. The difference in the source of clinical specimens in these two studies might indicate that the mechanisms of fosfomycin resistance are different in Taiwan and mainland China.

The vast majority of the MRSA isolates in the present study possessed at least one of *glpT* and/or *uhpT* mutations, implicating that the genes, encoding transporter mutants, contributed to fosfomycin resistance substantially. This result contrasted to the findings of the preceding study [15]. The prevailing subtype of mutations in the *glpT* and *uhpT* genes was also different from that reported by Fu et al. [15]. Forty-eight fosfomycin-resistant MRSA isolates with dual resistance mechanisms (*glpT* and *uhpT* mutations) belonged to *spa* type t002, again implying clonal spread of fosfomycin-resistant MRSA.

The most prevalent and the second most common *spa* types were t002 and t037 in our study, respectively, revealing that fosfomycin resistance in MRSA isolates were correlated to some spa types. A similar correlation was noticed in other countries, including Sweden, Korea, China, Iran, Africa, Canada, and Brazil [31]. An international, or even intercontinental spread of specific fosfomycin-resistant MRSA clones may be occurring.

In the present study, the susceptibility of the MRSA isolates to various antibiotics was similar to that reported in the previous studies from Taiwan [8, 27] but different from that in other countries [32–34]. Variation in drug susceptibility between geographic areas might be due to the presence of different prevalent MRSA clones and the difference in antibiotic selective pressure.

The major limitation of the present study is that it was conducted using the clinical MRSA isolates in Taiwan; thus, worldwide generalization of the results should be made carefully.

## Conclusions

In conclusion, our study illustrated that the fosfomycin resistance rate of the MRSA isolates increased significantly in the past, and mutations in the *glpT* and/or *uhpT* genes were key for inducing fosfomycin resistance. These findings indicated to physicians that they should prescribe fosfomycin cautiously for treating MRSA infections empirically. Furthermore, t002 was the most frequently seen *spa* type among the fosfomycin-resistant MRSA isolates. This was comparable to that in other countries globally. Therefore, it is necessary to continuously monitor fosfomycin resistance and its mechanisms.

## Supplementary information

**Additional file 1.**

## Data Availability

The datasets used during the current study are available from the corresponding author on reasonable request.

## Abbreviations

MRSA: methicillin resistant *Staphylococcus aureus*
TSAR: Taiwan Surveillance of Antimicrobial Resistance
PCR: polymerase chain reaction
*spa*: Staphylococcal protein A
MDRO: multidrug-resistant organisms
XDRO: extensively drug-resistant organisms
VRE: vancomycin-resistant enterococci
ESBL: extended spectrum beta-lactamases
CLSI: Clinical and Laboratory Standards Institute
