# Supplementary material for: Prevalence of fosfomycin resistance and gene mutations in clinical isolates of methicillin-resistant Staphylococcus aureus
Source: Antimicrob Resist Infect Control. 2020 Aug 17;9:135. doi: 10.1186/s13756-020-00790-x (PMC7430020; doi:10.1186/s13756-020-00790-x)
Supplement: Supplementary file 1 — Additional file 1. [file 13756_2020_790_MOESM1_ESM.pdf]

CERTIFICATE OF ENGLISH EDITING

This document certifies that the paper listed below has been edited to ensure that the language is clear and free of errors. The edit was performed by professional editors at Editage, a division of Cactus Communications.The intent of the author's message was not altered in any way during the editing process. The quality of the edit has been guaranteed, with the assumption that our suggested changes have been accepted and have not been further altered without the knowledge of our editors.

Title Of The Paper

Prevalence of fosfomycin resistance and gene mutations in clinical isolates of methicillin-resistant Staphylococcus aureus

Authors

Yi-Chien Lee, Pao-Yu Chen, Jann-Tay Wang, Shan-Chwen Chang

Job Code

SHTAI_445

Signature

Vikas Narang,
Senior Vice President,
Operations- Author Services, Editage

Date of Issue
September 20, 2019

Editage, a brand of Cactus Communications, of f ers prof essional English language editing and publication support services to authors engaged in over 500 areas of research. Through its community of experienced editors, which includes doctors, engineers, published scientists, and researchers with peer review experience, Editage has successf ully helped authors get published in internationally reputed journals. Authors who work with Editage are guaranteed excellent language quality and timely delivery.

Contact Editage

Worldwide	Japan	Korea	China	Braz il	Taiwan
request@editage.com	submissions@editage.com	submit-korea@editage.com	fabiao@editage.cn	contato@editage.com	submitjobs@editage.com
+1 877- 334- 8243	+81 03- 6868- 3348	1544- 9241	400- 005- 6055	0800- 892- 20- 97	02 2657 0306
www.editage.com	www.editage.jp	www.editage.co.kr	www.editage.cn	www.editage.com.br	www.editage.com.tw
